# Accessible Ecosystem for Clinical Research (Federated Learning for Everyone): Development and Usability Study

**DOI:** 10.2196/55496

**Published:** 2024-07-17

**Authors:** Ashkan Pirmani, Martijn Oldenhof, Liesbet M Peeters, Edward De Brouwer, Yves Moreau

**Affiliations:** 1 ESAT-STADIUS KU Leuven Leuven Belgium; 2 Data Science Institute Hasselt University Diepenbeek Belgium; 3 University Multiple Sclerosis Center Hasselt University Diepenbeek Belgium; 4 Biomedical Research Institute Hasselt University Diepenbeek Belgium

**Keywords:** federated learning, multistakeholder collaboration, real-world data, integrity, reliability, clinical research, implementation, inclusivity, inclusive, accessible, ecosystem, design effectiveness

## Abstract

**Background:**

The integrity and reliability of clinical research outcomes rely heavily on access to vast amounts of data. However, the fragmented distribution of these data across multiple institutions, along with ethical and regulatory barriers, presents significant challenges to accessing relevant data. While federated learning offers a promising solution to leverage insights from fragmented data sets, its adoption faces hurdles due to implementation complexities, scalability issues, and inclusivity challenges.

**Objective:**

This paper introduces Federated Learning for Everyone (FL4E), an accessible framework facilitating multistakeholder collaboration in clinical research. It focuses on simplifying federated learning through an innovative ecosystem-based approach.

**Methods:**

The “degree of federation” is a fundamental concept of FL4E, allowing for flexible integration of federated and centralized learning models. This feature provides a customizable solution by enabling users to choose the level of data decentralization based on specific health care settings or project needs, making federated learning more adaptable and efficient. By using an ecosystem-based collaborative learning strategy, FL4E encourages a comprehensive platform for managing real-world data, enhancing collaboration and knowledge sharing among its stakeholders.

**Results:**

Evaluating FL4E’s effectiveness using real-world health care data sets has highlighted its ecosystem-oriented and inclusive design. By applying hybrid models to 2 distinct analytical tasks—classification and survival analysis—within real-world settings, we have effectively measured the “degree of federation” across various contexts. These evaluations show that FL4E’s hybrid models not only match the performance of fully federated models but also avoid the substantial overhead usually linked with these models. Achieving this balance greatly enhances collaborative initiatives and broadens the scope of analytical possibilities within the ecosystem.

**Conclusions:**

FL4E represents a significant step forward in collaborative clinical research by merging the benefits of centralized and federated learning. Its modular ecosystem-based design and the “degree of federation” feature make it an inclusive, customizable framework suitable for a wide array of clinical research scenarios, promising to revolutionize the field through improved collaboration and data use. Detailed implementation and analyses are available on the associated GitHub repository.

## Introduction

### Real-World Data in Health Care: Toward Federated Learning Solutions

Real-world data (RWD), which encompass clinical and health data, are a fundamental asset in health care, providing invaluable insights for research and clinical applications [1,2]. However, for privacy, legal, collaborative, or practical reasons, RWD are typically *fragmented* into different data sources, such as electronic health records, patient registries, and other clinical repositories. This fragmentation, characterized by the dispersion of RWD across numerous health care systems and geographic locations, hampers the Findability, Accessibility, Interoperability, and Reusability (“FAIRness”) of the RWD [3]. Thus, the capacity to harness these fragmented data sources for evidence-based health care decision-making is hindered and must be improved [4,5].

While limited data sharing impedes the clinical research progress in general, the impact is particularly significant for research into low-prevalence diseases, where data scarcity already poses a challenge [6]. This issue becomes especially critical in urgent situations, such as the COVID-19 pandemic [7,8], where rapid data sharing is vital to quickly understand the impact of a novel disease.

Nevertheless, many challenges stemming from RWD fragmentation are systemic and can be seen as constraints that are difficult to eliminate. Legal data protection frameworks, ethical considerations, and organizational policies of different countries often limit the dissemination of such data both within and across countries [9-12]. These challenges are further complicated by different data registries using different data formats, standards, and infrastructures to reflect their specific needs [13]. This context raises the need for a global data sharing framework to facilitate the secure and efficient sharing of RWD, while accommodating most of the complexities of the clinical data ecosystem.

A key component of such a global data sharing framework is federated learning (FL), which offers a solution by training machine learning models on distributed data sets without centralization [14,15]. Nevertheless, current FL packages crucial for implementing this system fail to cater to the multifaceted requirements of health care stakeholders.

### Related Works

The FL framework ecosystem is characterized by its heterogeneity, prompted by contributions from both the open-source and industry communities, with each framework casted to address specific facets of FL’s inherent challenges. From enhancing security and privacy to ensuring scalability and promoting framework agnosticism to facilitating ease of use and customization, these frameworks collectively foster a conducive environment for the flourishing of FL applications.

Industry solutions such as IBM FL [16], the Clara Training Framework [17], and Sherpa.ai [18] offer specialized features but suffer from restricted accessibility due to their proprietary nature, limiting widespread application and innovation. Open-source FL frameworks, despite their appeal of broader accessibility, grapple with challenges unique to their design and intended use cases. Syft, also known as PySyft [19], in the context of offering advanced privacy-enhancing technologies, focuses on secure and private data analysis across various domains. Despite it emphasizes structured transparency systems, the complex setup process associated with Syft could impede its broad adoption. As a pioneering framework in the realm of privacy technology, Syft has undergone substantial revisions across its iterations. While aimed at improvement, these continuous overhauls may pose challenges for early adopters in terms of staying abreast with the framework’s evolution.

Frameworks such as FATE [20] and OpenFL [21] are distinguished by their strong emphasis on security and privacy, which are critical in sectors such as health care, where data confidentiality is essential. FATE is noted for its robust security and privacy features. However, it requires substantial infrastructural investment and has a steep learning curve, as there are some discrepancies in documentation clarity and availability across different parts of the framework. OpenFL also offers Intel-enabled trusted execution environment known as Safe Guard and mutual Transport Layer Security for all communications to ensure maximum privacy. However, implementing this framework on a broader scale is hindered and may necessitate specific hardware, software, or the activation of certain services [22]. TensorFlow Federated [23], while offering a specialized toolkit for TensorFlow models, limits its use to those committed to a singular modeling framework, potentially sidelining a broader audience that operates across diverse machine learning frameworks.

Flower [24] and FedLab [24] are recognized for their framework agnosticism and scalability. Flower emerges as a user-friendly and lightweight option, albeit still progressing through its developmental stages with real-world applications yet to be fully demonstrated. This reflects a common developmental trajectory among open-source FL frameworks, where achieving a balance between accessibility, ease of use, and broad applicability poses a significant challenge.

FedML [25] distinguishes itself through its versatility and recent architectural improvements, positioning it alongside frameworks such as Plato [26], known for supporting a diverse array of computing paradigms. This dual focus on facilitating FL research and the development of practical applications underscores its strengths. However, integrating significant updates may necessitate substantial maintenance for applications developed on its platform. Additionally, developers may need to reacquaint themselves with the framework’s updated features, presenting a potential challenge in adaptability and ongoing engagement.

Innovative approaches are evident in PaddleFL [27] and Galaxy FL [28], with Galaxy FL pioneering blockchain technology integration for improved data privacy and model confidentiality. This innovation broadens the FL framework landscape, introducing novel data protection and utilization aspects. However, blending FL with blockchain remains a debated topic within the literature, especially concerning the significant computation and communication overheads [29].

Lastly, FLSim [30] specializes in FL simulations, providing a comprehensive framework that incorporates differential privacy and secure aggregation scenarios. Similarly, Leaf [31] focuses primarily on simulation studies. While this specialization in simulation offers valuable insights, it may also narrow their applicability, potentially restricting their direct translation into real-world deployments.

For a more in-depth discussion of the FL frameworks, we guide readers to Multimedia Appendix 1, where Table S1 in Multimedia Appendix 1 [32-51] offers a comprehensive summary of the highlights and key features of various FL frameworks. Additionally, Table S2 in Multimedia Appendix 1 details the developmental progress and recent advancements within these frameworks, providing a clear snapshot of their evolution and current state.

### Navigating the Path Forward: The Need for an Accessible Ecosystem for Clinical Research

Beyond the challenges already covered, a recent review of FL in the biomedical domain reveals that only some research studies have provided reusable or reproducible source code alongside their research [52]. This highlights a common issue: the tendency to start a unique project for each separate data sharing initiative. This practice not only consumes valuable time and resources but also hinders the evolution of a universal ecosystem. Furthermore, the opportunity to build upon and contribute to an existing initiative is often overlooked.

Despite recent progress in the field, there is a critical demand for a more inclusive, user-friendly, and holistic framework in health care collaborative research [53,54]. A framework that empowers stakeholders from diverse backgrounds to fully engage in collaborative learning opportunities is essential. This requires FL solutions characterized by intuitive interfaces, streamlined workflows, comprehensive documentation, and a design process that actively seeks and incorporates feedback from a wide range of stakeholders. By prioritizing usability and inclusivity in developing FL solutions, we can significantly lower technical barriers. This approach enables wider participation and amplifies the contributions of collaborative health care research.

In this work, we present Federated Learning for Everyone (FL4E)—a dynamic, data-centric framework designed with inclusivity and adaptability at its core. FL4E is dedicated to efficiently converting RWD into actionable evidence, facilitating engagement from a broad spectrum of stakeholders, and enhancing collaborative learning experiences. Its hierarchical architecture offers a customizable data sharing ecosystem, facilitating a tunable balance between centralization and federation. With its modular approach, FL4E is general and provides an ecosystem-based environment that can support a wide range of collaborative research projects. This modularity further allows all stakeholders to contribute to the project by selectively engaging in the relevant part of the data sharing pipeline.

### Key Contributions

- We propose a unique FL framework designed to cater to the diverse needs of health care stakeholders and to adapt to various study designs. This design is further enhanced by offering a tunable balance between centralization and federation.
- We formalize this balance by introducing the concept of “degree of federation.”
- Our evaluation of the framework, using 2 real-world data sets, underscores the value of federation. Specifically, hybrid experiments conducted at different degrees of federation deliver performance comparable to fully federated setups.

## Methods

### Overview

In this section, we rigorously investigate the fundamental principles underlying FL4E, systematically articulating its core concepts. This entails a meticulous examination of the pivotal modules and essential components that constitute FL4E, with a particular focus on their functionalities and interrelationships. Moreover, this section highlights the architectural nuances of FL4E, comprehensively detailing its structural configuration and the dynamics of its operational framework.

### Key Concepts of FL4E

First, the concept of “degree of federation” within the FL4E framework introduces a novel, adaptable, and pragmatic approach to collaborative data analysis designed to meet stakeholders’ varied needs and limitations. This feature enables participants to customize their level of involvement and data sharing based on their specific infrastructural capacities, regulatory requirements, and privacy considerations.

FL4E acknowledges that while federated models offer significant privacy advantages, their practicality may vary due to different constraints. Therefore, FL4E is designed to accommodate various levels of participation for stakeholders, ensuring flexibility if one data sharing stream proves unfeasible.

Therefore, the choice between a centralized or federated model within FL4E is presented not as a dichotomy but as a continuum. On the one hand, centralization may simplify operations and enhance efficiency, but it could compromise privacy to some degree. Conversely, full federation prioritizes privacy, possibly adding operational and analytical complexity. FL4E’s design empowers stakeholders to find a balance that best suits their needs, governance frameworks, and regulatory environments.

**Figure 1:**
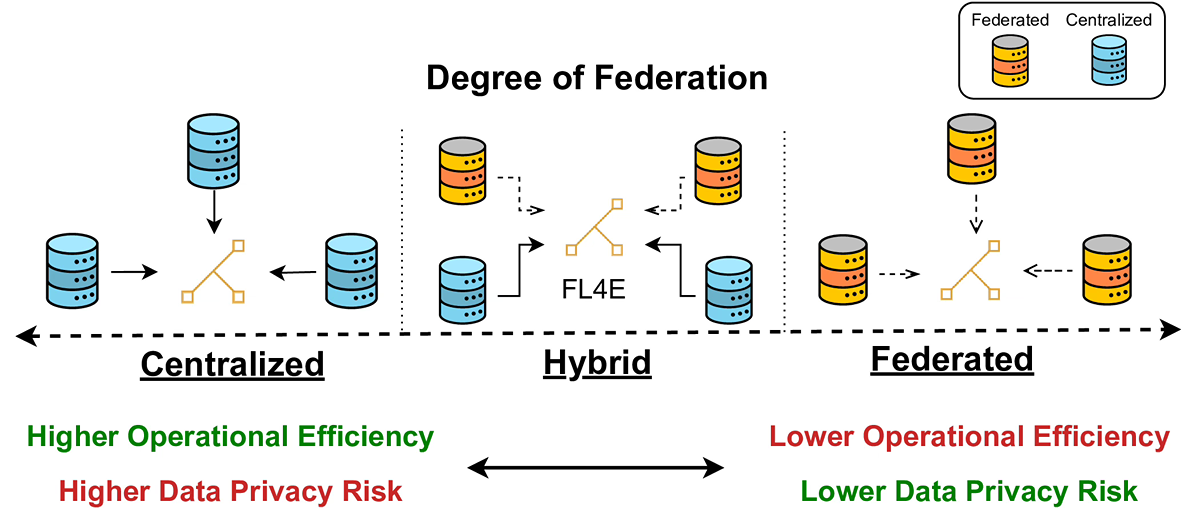
The degree of federation characterizes the balance between fully centralized and fully federated setups, leading to hybrid solutions where some of the stakeholders centralize their data while others prefer a federated approach. FL4E: Federated Learning for Everyone.

This adaptable strategy has proven effective in accommodating diverse data sharing needs, as demonstrated by the Global Data Sharing Initiative for Multiple Sclerosis and COVID-19 [55,56]. The deployment of a versatile hybrid data acquisition system, merging traditional data sharing with federated methods, has not only broadened inclusivity and flexibility but also led to a notable (3527/7757, 45.47%) increase in data collection volume compared to initial efforts [57]. These results underscore the “degree of federation” practical value, showcasing the advantages of integrating such adaptability into data acquisition and sharing frameworks. Different configurations within the degree of federation are depicted in Figure 1.

However, it is essential to emphasize that the framework fosters regulatory compliance across all participation methods. Whether stakeholders prefer the simplicity and direct oversight of a more centralized model or the enhanced privacy of a federated approach, stakeholders are reminded of their collective obligation to adhere to prevailing data protection regulations.

Second, ecosystem-based collaborative learning: FL4E framework introduces a paradigm shift from conventional project-specific FL to an “ecosystem-based” approach, underscoring a strategic pivot toward creating a unified, adaptable platform tailored for conducting various analyses centered on specific diseases or health conditions. At the heart of the “ecosystem-based” methodology is its holistic perspective, aiming to formulate a comprehensive and synergistic environment for data sharing and collaborative research.

An ecosystem, in this context, is envisioned not just as a collection of data or series of disjointed projects but as a dynamic, collaborative network of stakeholders. This includes researchers, health care professionals, patients, and policy makers, all united by a common goal: understanding and addressing a particular health issue. By fostering such an ecosystem, FL4E encourages the pooling of diverse resources, expertise, and perspectives. This collaborative efficiency breaks down traditional silos, allowing for more efficient use of resources through shared data and findings.

Moreover, encompassing a wide array of stakeholders and research questions enables the ecosystem to provide a more nuanced and comprehensive understanding of the health condition under investigation. The diversity of perspectives and expertise enriches the research outcomes, offering holistic insights far beyond what could be achieved in isolation.

This approach is inherently adaptable and capable of evolving in response to new research findings, emerging health challenges, and shifts in the regulatory landscape. Such flexibility ensures the long-term sustainability and relevance of the ecosystem, enabling it to continue generating valuable insights over time.

Regarding its structure, FL4E features a nested design. Within each ecosystem, several studies are carried out, each focusing on specific research questions related to the overarching theme. These studies, while operating under their unique regulatory and consent protocols, incorporate the degree of federation. They can include a data set, data quality scripts, and analyses tailored to their specific research queries.

**Figure 2:**
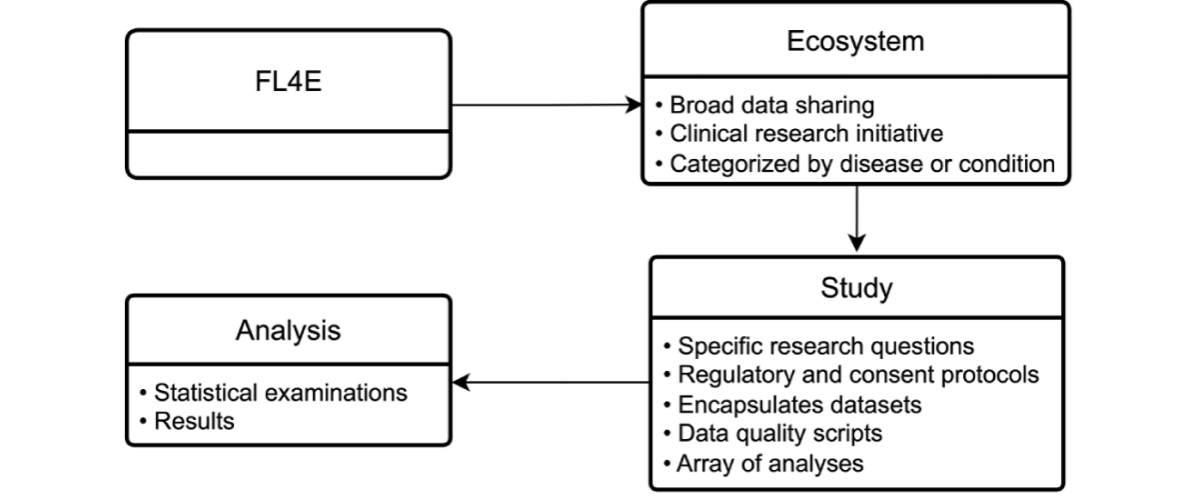
FL4E’s nested approach schema: the schema presents the ecosystem approach of the FL4E, facilitating the incorporation of diverse studies framed by different research questions. It further highlights the support for a wide range of analyses, leading to the execution of statistical experiments. FL4E: Federated Learning for Everyone.

FL4E’s structure also incorporates simplicity and accessibility in its design. The intuitive user interface encourages contributions from stakeholders, fostering a collaborative environment that is integral to the ecosystem’s success. This systematic approach to collaboration and data sharing is visually depicted in Figure 2.

The FL4E framework builds upon the foundational concepts of the Flower framework [32]. Flower’s flexibility, support for multinode execution, and agnosticism to both machine learning frameworks and programming languages significantly contribute to the overall adaptability and versatility of FL4E.

### Modules and Functionality

#### Overview

**Figure 3:**
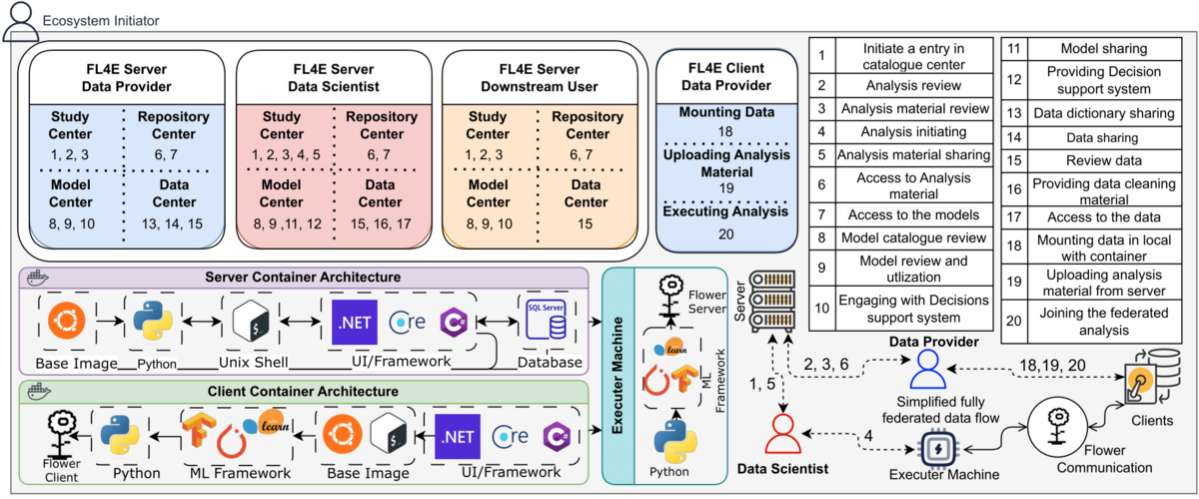
The high-level architecture of FL4E: This figure showcases a comprehensive framework designed to accommodate the nuanced interactions of diverse stakeholders. The architecture includes the expected user stories across 3 primary categories of participants: data providers, data scientists, and downstream users. Each stakeholder group engages with FL4E platform through distinct pathways. The architecture diagram features a table outlining the unique interactions of the stakeholders, mapping their respective roles and activities within the FL4E framework. This detailed mapping clearly explains how each stakeholder contributes to and benefits from the FL4E, highlighting the platform's versatility and user-centric design. At the core of our architecture lie 3 fundamental components: the server, client, and executor machine, each vital to the execution of FL tasks. The diagram we provide elucidates their interconnected roles, showcasing the seamless flow of data, scripts, and analytical results across the system. The server acts as the orchestrator for FL tasks, hosting the primary web application within a Docker container as an ASP.NET application. It securely manages the platform's data, housed on an SQL server in a dedicated hosting environment. This component is crucial for coordinating tasks and uses a Python environment to manage secure data sharing and preprocessing of “data center” module of the framework. On the client side, implemented as a Docker-based image, it runs on the data contributor's machine. This component is essential for integrating RWD into the FL process. Developed using Python and ASP.NET for web applications, the client-side component establishes a connection to the executer machine. On the other hand, the executor machine plays a crucial role in conducting the analysis. It is designed to receive client updates. This adaptable component configuration allows data scientists to tailor it according to their specific analytical needs and preferences. FL: federated learning; FL4E: Federated Learning for Everyone; RWD: real-world data.

This section provides an in-depth overview of the core modules within the FL4E framework: the Study Center, Repository Center, Model Center, and Data Center. Each module’s role and anticipated functionality are detailed, showcasing how the division into distinct centers contributes to effectively deploying the overarching ecosystem. Figure 3 complements this by visually representing the architectural arrangement of server and client components. By detailing the anticipated interactions among stakeholders and illustrating the operational flow within the framework, the architecture provides a solid foundation for understanding the potential and practical application of FL4E in advancing health care data-driven research and insights.

#### Study Center

The Study Center is a critical piece in the ecosystem design of the framework. It coordinates research activities via the Study Catalogue and the Analysis Center, highlighting the interconnectivity and synergy within the system.

The Study Catalogue functions as a cooperative repository where various stakeholders can share and access overarching information or metadata about different studies. Collaborations can be initiated by a data scientist with an intriguing research question but needing more data or by a data provider possessing the required data but lacking the analytical tools. Downstream users can further enrich the catalogue by contributing scientific articles or proposing research questions relevant to other stakeholders. Any new entry to the catalogue should include the title, a brief description, the study lead or author, and the current status of the entry (ie, active or not). If the entry involves any data, this should also be noted, as the data would be subsequently managed within the Data Center.

In contrast, the Analysis Center is primarily designed for data scientists. Here, they can create an analysis entry derived from a study catalogue record. While doing so, they must furnish comprehensive details, including the exact analysis title, a descriptive summary, and the privacy status of the analysis (private or public). In a private setting, data scientists can selectively grant access to specific users (data providers). This means only authorized users can access these entries.

In the FL4E framework, data scientists play a crucial role in orchestrating the analysis by providing essential scripts for client-side execution. Given that the FL4E backend is built upon the Flower framework, these scripts must align with this framework’s requirements. The machine learning–agnostic nature of this foundation offers a substantial flexibility to data scientists, allowing them to craft these scripts within the ambit of their expertise and the specific needs of the analysis. This flexibility is further augmented by Flower’s support for secure channels, enabling the addition of privacy-preserving configurations to the analysis execution. The integration of Flower’s capabilities within FL4E not only simplifies the technical implementation of FL projects but also empowers data scientists to innovate and experiment with various analytical models and privacy configurations. This approach ensures that FL4E remains a versatile and secure platform for FL, accommodating a wide range of research objectives and data privacy standards.

To ensure transparency, the process of sharing scripts among the stakeholders is not automated. Clients may review and, if agreed, run these scripts locally using the FL4E-client component. In keeping with the principles of modularity and customization, the server does not include a built-in compiler to execute the analysis. Data scientists should disclose the IP and port address of the designated machine to data providers for analysis execution, enabling FL process participation. It is vital to note that no analysis should proceed in the Analysis Center without explicit consent from participating centers. If a particular study is associated with any data on central server, data scientists should incorporate the data as a centralized client into the federated analysis, effectively acting as an additional presumptive federated client in the data scientist’s machine.

#### Repository Center

The Repository Center serves as a warehouse for all scripts and models essential for stakeholders’ research and collaboration. It serves both as a repository to disseminate the scripts and models to the relevant stakeholders, and as an archive that stores past analyses and provides examples facilitating the creation of new studies. It seamlessly integrates with the Study Center and Model Center, enabling direct and organized access to all shared scripts and models across these centers.

#### Model Center

The Model Center is designed to facilitate the coordination and sharing of trained models. Similar to the Study Center, the Model Center is structured around 2 main elements: the Model Catalogue and the Model Repository.

The Model Catalogue serves as a database for high-level information about various trained models. This can include the title, description, type of model, study lead, and status. The Model Catalogue is particularly useful for downstream users and data scientists who come across an interesting model and wish to share it within the ecosystem, or for those who want to share the outcome of an analysis conducted within the Study Center.

Furthermore, the Model Repository is where detailed information about each trained model is stored. This includes the relationship of the model to a specific Model Catalogue entry or Analysis Center entry. Like the Study Center, the Model Center also adopts authorization layers to ensure that only authorized users can access certain models. A typical scenario is a trained model that would only be shared among the participants that contributed with data.

However, if needed, the Model Center can also be used to disseminate a model more broadly. Indeed, users can attach trained models to the results of an analysis. This means that a user who may not have been able to join an analysis can still benefit from the trained models, download them, and test them on their local data. This is especially useful for data providers who want to validate models against their data sets or for data scientists who wish to use them as pretrained models in a similar domain.

Furthermore, the Model Center allows data scientists to provide a decision support system as a result of a trained model. They can inject an HTML web page into the Model Repository entry, serving as a web-based dashboard. This user-friendly interface enables downstream users, even those without expert knowledge, to engage with the system effectively.

#### Data Center

The Data Center plays a crucial role in actualizing the concept of the “degree of federation” within the FL4E framework. Beyond providing data sharing capabilities, it also enables data integration and aggregation, meaning this module stores the data in a central server. Data providers and data scientists are central to the sharing process within the Data Center, using a meticulous 3-step routine to ensure data quality, along with effective data sharing and aggregation. The first step is (1) the raw data sharing scheme, followed by (2) the data cleaning and enhancement, and (3) the data sharing and aggregation.

#### Raw Data Sharing Scheme

The process initiates when the data provider shares a raw data scheme via a data dictionary, a static file describing the data structure. This dictionary must be attached to an existing study catalogue entry along with details like title, description, and data type (public or private). For private data, the provider must list authorized users.

#### Data Cleaning and Enhancement Script

Once the data scheme is established, data scientists must create a Python script for data cleaning and enhancement. This script ensures data quality by handling duplicates, missing values, and validating value ranges based on information of the data schema. This script should be uploaded to the Data Center and linked to the data dictionary.

#### Data Sharing and Aggregation

After setting up the data dictionary and cleaning script, data sharing can occur. Each data share triggers the cleaning script execution, with the outcomes appended to a core dictionary and each upload saved as a distinct file. This setup permits various data providers to share data in line with the data dictionary, with the script guaranteeing aggregation into a single file. Data scientists can then access this aggregated file for analytical use if access is granted to them.

### Implementation of the Framework

The FL4E framework is divided into server-side and client-side implementations. Server-side, the framework is a microservice-based web application, designed using ASP.NET Core [58]. It adheres to the N-tier architecture and uses the Model-View-Controller design, along with a repository pattern, leading to a scalable and maintainable application. The application is segmented into 4 layers: Application, Model, Utility, and Data Access. Each layer performs distinct tasks, such as managing user authorization, defining data structures, handling requests, and interacting with the database. ASP.NET Core Identity module is used for authentication and authorization, while an admin control panel supervises user registrations in the interface layer of the framework [58].

Client-side, the framework aims for simplicity and ease of use, also leveraging the ASP.NET Core framework. It incorporates 3 primary interfaces for data mounting, script uploading, and script execution. The data mounting interface facilitates user selection and mounting of data files; the script uploading interface enables users to upload scripts acquired from the server; and the script execution interface allows for the running of these scripts, using the Flower framework to secure the communication between the client and server.

Ensuring the seamless operation and accessibility of the FL4E platform, both the client and server components are encapsulated as Docker containers. This strategic choice underpins the platform’s commitment to ease of deployment, consistency across different environments, and portability, thereby, significantly enhancing the user experience for participants in FL projects.

A fully functional prototype of the FL4E server component has been successfully deployed to Microsoft Azure, showcasing the platform’s robustness [59]. This deployment not only demonstrates FL4E’s capability to leverage cloud infrastructure but also its readiness to support FL projects.

To further acquaint potential users and stakeholders with FL4E’s functionalities and user interface, a comprehensive video demonstration has been made available [60]. This visual guide serves as an invaluable resource for understanding the platform’s operational flow, features, and the simplicity of initiating and participating in FL studies.

For those interested in a deeper exploration or customization of FL4E, the source code for both the server and client components is readily accessible [61]. This openness ensures transparency, fosters a community of collaboration, and enables continuous improvement of the platform by allowing researchers, developers, and data scientists to contribute to its development.

### Ethical Considerations

This study conducted secondary analyses of openly licensed repository data sets for demonstration purposes in the results section. No sensitive data were used for the cocreation of the framework. All data set sources are cited and adhere to open licensing agreements. For more detailed information about the data sets, please refer to the original sources.

## Results

### Data Sets

We evaluated our framework using 2 real-world clinical data sets from FLamby [62]: Fed-Heart-Disease and Fed-TCGA-BRCA. The Fed-Heart-Disease data set contains 740 records from 4 centers, detailing 13 clinical features and a binary heart disease indicator for each patient. We used a logistic regression model for prediction. The Fed-TCGA-BRCA data set, sourced from The Cancer Genome Atlas’s Genomics Data Commons portal, has data from 1088 patients with breast cancer across 6 centers. With 39 clinical features and each patient’s time of death, we used a Cox model to predict the risk of death.

Additionally, the Wisconsin Breast Cancer data set was incorporated into the GitHub repository as an extra use case [61,63]. It serves as a set of foundational scripts that can be generalized and act as guidelines for hybrid experiment settings in the FL4E framework.

### Degree of Federation Scenarios

To demonstrate how FL4E adapts to various research questions, and how it modulates the degree of federation, we conducted 3 experiments using these data sets:

#### Fully Federated Experiment

In this scenario, every client participates in the FL process with each one contributing to the global model using their respective local data sets. This scenario aligns with the conventional FL setup. The data flow for this experiment is depicted in Figure 3.

#### Hybrid Experiment

In the hybrid setup, while all clients are part of the training process, some of them contribute their data centrally to the server. For instance, with the Fed-Heart-Disease data set, clients 1 and 2 were randomly selected to centrally contribute their data, while the rest participated as federated clients. For the Fed-TCGA-BRCA data set, clients 2, 3, and 5 joined as centralized clients.

#### Centralized Experiment

For this experiment, all clients send their data to the central server, and the model is trained centrally. This design aims to provide a benchmark for comparing the performance of the federated and hybrid approaches with the centralized model.

### Experimental Findings

We evaluated our FL models using area under the receiver operating characteristic curve (ROC-AUC) and accuracy metrics for the Fed-Heart-Disease data set and the concordance index for the Fed-TCGA-BRCA data set. These metrics were chosen according to their ability to assess model performance in binary classification and survival analysis task, while accounting for potential class imbalance. For federated experiments, we implemented multiple FL strategies: FedAVG [14], FedOpt [64] (including FedAdagrad and FedYogi), and FedProx [65]. Hyperparameter tuning was performed using grid search on a centralized setting and applied to each experiment of the data sets. To ensure consistency, each experiment was repeated 5 times. The results of these experiments are summarized in Table 1.

In our analysis of the Fed-Heart-Disease data set, as illustrated in Multimedia Appendix 2 and detailed in Table 1, notable behavior patterns emerge from clients 2 and 3. These patterns indicate the effects of class imbalance and quantity skew, suggesting that smaller clients with imbalanced data sets may benefit more from participating in an FL environment than operating in isolation. This insight is harmonious with the foundational principles of the FL paradigm, which aspires to harness the potential of diverse data sources to enhance model robustness and generalizability.

To clarify the evaluation process, we introduce the notation where *K* represents the total number of clients participating in the FL process, and *n_i_* denotes the size of the data set for the *i*-th client, where *i* ranges from 1 to *K*. Furthermore, *E_i_* signifies the evaluation metric (such as accuracy or ROC-AUC) obtained from assessing the global model by the *i*-th client. The sum of the data set sizes across all clients is represented by *N*, calculated as 
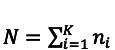
. Therefore, the overall evaluation metric *E* for the global FL model is calculated using the formula 
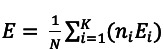
, offering a comprehensive measure of performance across the federated network.

This approach significantly impacts the evaluation process by prioritizing the characteristics of individual clients over the generalizability of the FL model. This provides a more comprehensive understanding of model performance across diverse environments. However, the potential for skewness in federated evaluation, often stemming from imbalanced clients, arises from its reliance on a weighted average of individual client metrics. This introduces challenges, particularly when directly comparing it to centralized performance.

It is important to note that for a direct comparison between federated and centralized evaluations, the ideal scenario involves assessing the FL model using a separate global test set on the server side. While this approach aligns more closely with the methodology used in centralized evaluations, it is not always feasible, especially in real-world settings where maintaining a separate test set on the server can be impractical due to data governance and privacy concerns.

Contrary to initial expectations that centralized models would outperform others, our analysis unveiled that federated and hybrid models exhibit superior performance based on the ROC-AUC measure, even though the accuracy metrics might suggest a different narrative. To ensure the reliability and consistency of our findings, we have conducted rigorous comparisons with the original benchmark [62]. These comparisons demonstrate that our observations, especially concerning the performance metrics of centralized and federated models such as accuracy, are in line with existing research. This alignment reinforces the validity of our analysis despite the inherent challenges in conducting direct comparisons between federated and centralized performance evaluations [62].

Similarly, the Fed-TCGA-BRCA study substantiates the advantage of FL. The heterogeneity of clients in the data set analysis, as highlighted by FLamby [62], indicates that this diversity significantly influences training performance, underscoring the potential benefits of FL.

Notably, the hybrid experiment yields a crucial insight: it offers performance that matches fully federated models while significantly outperforming local settings for smaller clients. Importantly, this is achieved with considerably less complexity and overhead associated with managing federation across multiple data centers. This setup enables centralized management of in-house data, providing analysts with direct access (within regulatory boundaries) and flexibility for advanced analyses. By having data information at their disposal, analysts can develop more effective scripts, enabling advanced analyses with greater flexibility.

**Table 1:** Experiments carried out using FL4E^a^ with the Fed-Heart-Disease and Fed-TCGA-BRCA data sets, demonstrating adaptability to varying degrees of federation.

Experiment and parameter		Fed-Heart-Disease, mean (SD)			Fed-TCGA-BRCA, mean (SD)
		ROC-AUC^b^	Accuracy	C-index^c^	
**Fully federated**					
	FedAvg	0.846 (0.002)	0.733 (0.007)	0.732 (0.030)	
	FedAdagrad	0.841 (0.029)	0.726 (0.05)	0.748 (0.016)	
	FedYogi	0.803 (0.051)	0.715 (0.032)	0.745 (0.037)	
	FedProx	0.846 (0.003)	0.741 (0.006)	0.725 (0.007)	
**Hybrid experiment**					
	FedAvg	0.825 (0.004)	0.740 (0.054)	0.656 (0.06)	
	FedAdagrad	0.821 (0.008)	0.741 (0.005)	0.776 (0.036)	
	FedYogi	0.794 (0.013)	0.710 (0.039)	0.726 (0.041)	
	FedProx	0.822 (0.004)	0.737 (0.012)	0.439 (0.227)	
**Local experiment**					
	Client 0	0.842 (0.009)	0.753 (0.011)	0.668 (0.064)	
	Client 1	0.882 (0.007)	0.800 (0.013)	0.445 (0.237)	
	Client 2	0.546 (0.271)	0.550 (0.199)	0.635 (0.166)	
	Client 3	0.542 (0.054)	0.559 (0.096)	0.570 (0.140)	
	Client 4	—^d^	—	0.851 (0.078)	
	Client 5	—	—	0.666 (0.001)	
	Clients 1 and 2	0.819 (0.003)	0.752 (0.008)	—	
	Clients 2, 3, and 5	—	—	0.578 (0.125)	
Centralized		0.812 (0.003)	0.753 (0.007)	0.609 (0.207)	

^a^FL4E: Federated Learning for Everyone.

^b^ROC-AUC: area under the receiver operating characteristic curve.

^c^C-index: concordance index.

^d^Not applicable.

## Discussion

FL4E presents a novel approach to collaborative learning in a health care context, by innovatively balancing centralization and federation. This allows health care organizations to engage in FL according to their infrastructure and policy capabilities. Composed of the Study Center, Repository Center, Model Center, and Data Center, FL4E’s modular, ecosystem-oriented design caters to a wide variety of stakeholders, thereby facilitating multiparty clinical research collaborations.

However, FL4E is not alone in promoting deployment ease and inclusivity in FL environments. FeatureCloud [66] advances FL by offering a platform that simplifies deployment across health care and research sectors. Aimed at democratizing multi-institutional analyses, FeatureCloud ensures that advanced programming skills or complex setups do not impede participation. The AI Store module, facilitating algorithm sharing and access, highlights FeatureCloud’s commitment to broadening FL adoption.

In parallel, the emergence of UniFed [67] underscores a similar commitment to simplifying FL’s application by unifying diverse open-source frameworks. UniFed’s approach standardizes FL experimentation and deployment, offering a configuration-based, schema-enforced task specification that eases the distributed execution of FL projects. By supporting various FL frameworks, UniFed addresses the challenges of workflow diversity, data format discrepancies, and interpretation variations, thereby streamlining the FL experimentation process.

What distinguishes FL4E in the evolving landscape is its focus on the “degree of federation” and the adaptability it brings, ensuring the framework is inclusive, adaptable, and can host hybrid experiments. Unlike FeatureCloud, which was developed from scratch, FL4E builds on the well-regarded Flower framework, providing a robust, accessible foundation and using this base, an adaptive, ecosystem-based model, designed to cater to diverse stakeholder preferences and requirements. FL4E extends the democratization vision of FL, incorporating a modular, customizable design that enhances engagement and ensures versatility across various applications. Additionally, FL4E adheres to key coding principles such as N-tier architecture and the repository pattern. These design choices promote ease of use, flexibility for modifications, and future-proofing the framework, further distinguishing FL4E by making it more accessible, maintainable, and adaptable to advancements in FL.

As the FL field continues to mature, platforms such as FeatureCloud and UniFed play crucial roles in lowering technical barriers and fostering an environment conducive to collaborative innovation. Their contributions, along with FL4E’s nuanced understanding of stakeholder engagement, collectively propel the FL ecosystem toward a future where data-driven insights are accessible to a broader spectrum of the health care and research community, thereby amplifying the impact of FL in advancing health care outcomes and research endeavors.

Nonetheless, FL4E still presents some limitations. Version control can pose challenges due to the Flower package dependency, and significant version changes may require internal updates. Moving from technical concerns to regulatory issues, ensuring adherence to regional data privacy regulations necessitates diligence from initiators and study leads, who must establish proper agreements for data sharing. Additionally, the framework’s dependence on manual communication among stakeholders could potentially impede its universal accessibility. This last point is particularly critical, as the system’s effective functioning heavily relies on the presence of a pre-established network of collaborators where members have a certain level of familiarity or knowledge of each other.

Although FL4E and other FL systems offer potential technical solutions to collaborative learning in health care, the influence of societal barriers should not be underestimated. Hence, continuous education, stakeholder engagement, and proof-of-concept demonstrations within controlled environments are crucial for successful deployment.

Future development path to FL4E, the demand for advanced privacy measures becomes increasingly apparent. While FL introduces a novel approach to leveraging patient-level data across decentralized sources, it is essential to recognize that this method is not without its vulnerabilities [68-70]. The process of sharing impersonal statistics during FL can inadvertently encode sensitive information, thus introducing potential risks and challenges to data privacy [68,71,72].

The architecture of FL, which is designed to enhance privacy by eliminating the need for direct data sharing, is nevertheless susceptible to sophisticated attacks and privacy breaches. These vulnerabilities include inference attacks, where malicious entities may attempt to reconstruct personal data from model updates, as well as direct cyber threats that target the integrity and confidentiality of the FL process [68]. The body of literature on FL privacy highlights the critical importance of acknowledging these risks, with studies identifying various attack vectors that could potentially compromise the privacy guarantees of federated model sharing [72].

Therefore, despite FL’s significant reduction in the risk of direct data exposure, it introduces new complexities in safeguarding against indirect data inference and cyberattacks. This nuanced understanding necessitates a cautious approach in the implementation of FL, particularly when handling sensitive health care information. The design of FL workflows, including the development of infrastructure and study plans, must incorporate robust security measures and privacy-preserving techniques.

To this end, it is crucial to leverage the latest advancements in secure computing, differential privacy, and encryption technologies to strengthen the FL process against potential vulnerabilities [73-75]. Acknowledging these risks and adequately preparing for them is paramount, ensuring that the advantages of using decentralized data through FL do not come at the expense of patient privacy or data security. The implementation of industry-scale privacy measures is critically important in FL applications, as emphasized by recent studies [74]. However, these measures must be carefully balanced to ensure that data protection does not compromise the computational efficiency of FL analysis.

To ease deployment, recent research in practical FL points toward promising techniques, such as soft computing’s fusion with FL [76] and the use of FL in cloud-edge collaborative architectures [77]. Such strategies can address concerns over data privacy, high communication costs, and data management difficulties, offering a promising direction for future investigations.

While the manual steps (for instance, script exchanges) in FL4E are designed for transparency, automating these steps could improve the framework’s adaptability and usability. Additionally, transitioning from the current ASP.Net to Python could amplify the framework’s appeal, especially given Python’s strong foothold in the data science realm. As health care data expand in volume and complexity, frameworks such as FL4E, with further advancements and community involvement, stand to be vital tools in catalyzing transformative progress and collaboration in the domain.

## Appendix

Multimedia Appendix 1 Landscape analysis: prominent frameworks for federated learning.

Multimedia Appendix 2 Delineating client heterogeneity in real-world applications: exploration of class imbalance in the Fed-Heart-Disease analysis.

## Abbreviations

FL: federated learning
FL4E: Federated Learning for Everyone
ROC-AUC: area under the receiver operating characteristic curve
RWD: real-world data

## Data Availability

The data sets used for the analysis purposes of this study are available at the FLamby repository on GitHub [78]. FLamby serves as a benchmark for cross-silo data sets for federated learning applications, with a focus on health care application [62].
