# Supplementary material for: Accessible Ecosystem for Clinical Research (Federated Learning for Everyone): Development and Usability Study
Source: JMIR Form Res. 2024 Jul 17;8:e55496. doi: 10.2196/55496 (PMC11292148; doi:10.2196/55496)
Supplement: Multimedia Appendix 1 [file formative_v8i1e55496_app1.docx]

**Multimedia Appendix 1.** Landscape analysis: prominent frameworks for federated learning.

A rich diversity marks the landscape of FL frameworks, each offering a unique integration of features tailored to address the multifaceted challenges of distributed, privacy-preserving machine learning. A methodical investigation uncovers a range of design principles, varying from those placing a high priority on security and privacy to frameworks that emphasize scalability, platform neutrality, and user-friendliness. This analysis endeavors to cover this landscape, highlighting the intrinsic attributes, potential limitations, and the distinct application domains of these frameworks, thereby providing a comprehensive overview.

Table S1 in Multimedia Appendix 1, offers an insightful comparison across various FL frameworks, shedding light on their distinctive features, limitations, key application areas, and their status as either open-source or proprietary tools. This comparative analysis reveals the rich diversity within the FL ecosystem, demonstrating how each framework is uniquely positioned to address specific needs and challenges.

This compilation highlights the inherent trade-offs involved when selecting an FL framework, such as balancing ease of use against computational demands or gauging the level of community support available.

**Table S1.** Comparison of federated learning frameworks: an overview of features, limitations, and application domains.

| Framework | Core/Key Features | Limitations/Remarks | Specific Use Cases/Applications |
| --- | --- | --- | --- |
| PySyft [33], [34] | - Enhances privacy in data processing, combining PETs for structured transparency systems. - Supports homomorphically encrypted operations and split neural networks for inference. | - Aims to enable secure and private data analysis across domains, introducing Duet for privacy through encryption and controlled data access. | Secure and private data analysis across various domains, supporting structured transparency in data flows. |
| FATE [35], [36], [37] | - Industrial-grade, supports various FL scenarios and algorithms. - Modular, scalable, with a strong emphasis on security and privacy through homomorphic encryption and MPC. - Backed by a robust community and ecosystem. | - Complexity and steep learning curve for new users. - Deployment challenges, especially in large-scale environments. - Significant resource requirements. | Suitable for industries where data security and privacy are paramount, such as finance and healthcare. |
| FedML [25], [38], [39] | - Versatile, supporting a wide range of domains including NLP, CV, and IoT. - Flexible and customizable architecture with comprehensive benchmarks. - Promotes open and collaborative development. | - Complexity for beginners. - Resource intensity for full utilization. - Dependent on active community engagement. | Broad spectrum of FL research and applications, from edge devices to cloud-based environments. |
| Flower [67], [40] | - Highly customizable and extendable. Framework agnostic, supporting integration with popular ML libraries. - Scalable, tested with over 10,000 clients. - Focuses on both academic research and practical application. | - Potential integration complexities and scalability challenges in very large setups not specifically mentioned. | Projects requiring integration with various ML frameworks and scalability, from mobile to edge devices. |
| TensorFlow Federated [23] | - Two-layer architecture for custom federated algorithms. - Encourages research and experimentation. - Support for non-learning computations for federated analytics. | - Requires familiarity with TensorFlow for optimal use. - Design choices may limit flexibility with non-TensorFlow technologies. | Developing privacy-preserving models like mobile keyboard prediction without central data collection. |
| FederatedScope [41] | - Modular and extensible design, supporting a broad range of FL applications. - Unique event-driven architecture for flexible and powerful asynchronous federated training. - Comprehensive documentation and tutorials. | - Ease of use for new users enhanced by extensive documentation, though specific challenges are not mentioned. | Varied FL applications, from CV and NLP to graph FL. |
| OpenFL [21], [22], [42] | - Scalable, capable of managing large federations. - High emphasis on security, employing Intel-enabled TEEs. - Designed for ease of use with support for popular ML frameworks. | - Setup and familiarity with security features required for optimal use. | Expansive FL projects requiring high security, such as healthcare data analysis. |
| FedLab [24], [43] | - Modular design, supporting diverse data distributions and federated algorithms. - Comprehensive baselines and data partitions for research. | - Effectiveness in highly heterogeneous environments or under specific operational constraints could vary. | Research focusing on FL under realistic conditions, addressing IID and non-IID settings. |
| MetisFL [44] | - Adheres to principles of modularity, extensibility, and configurability. - Supports TensorFlow and PyTorch, extendable to other backends. - Application domains span CV, NLP, and more, reflecting its general-purpose, domain-agnostic nature. | - Challenges with very specific or niche requirements might arise, not explicitly mentioned. | General-purpose applications across various domains such as CV and NLP. |
| NVFlare [45] | - Focuses on privacy preservation. - Supports popular ML/DL frameworks. - Provides tools for secure provisioning, orchestration, and monitoring. | - Implementation challenges in domain-specific applications might be encountered. | Secure, privacy-preserving collaborations in AI research fields across distributed setups. |
| IBM FL [16] | - Highly configurable and extensible, supporting a wide range of ML techniques. - Includes a comprehensive library of fusion algorithms. | - Significant setup and configuration might be required. - Broad applicability with potential for complex deployments, though specific limitations are not discussed. | Enterprise environments with needs for data privacy in supervised, unsupervised, and reinforcement learning projects. |
| PaddleFL [27], [46] | - Supports horizontal and vertical FL strategies. - Leverages PaddlePaddle for distributed training and Kubernetes for deployment. | - Preparing to extend support for vertical FL and deployment schemes, indicating ongoing development and optimization. | Applications in CV, NLP, and recommendation systems, addressing data isolation and secure data knowledge sharing. |
| FedScale [47] | - Offers realistic FL tasks and scalable runtime with diverse datasets. - Focuses on benchmarking model and system performance at scale. | - Aims to address client heterogeneity and large-scale FL deployment challenges, reflecting a comprehensive approach to FL benchmarking. | Facilitates reproducible FL research across domains like image classification and speech recognition, aiming for heterogeneity-aware optimizations. |
| Plato [26], [48] | - Designed for scalable and reproducible FL research. - Supports a wide range of FL algorithms, scalable, and extensible. | - Focuses on scalability, extensibility, real-world system implementations, and reproducibility, suggesting broad applicability. | Scalable and reproducible FL research, supporting deployment in various environments. |
| EasyFL [49] | - Low-code platform for FL experimentation and prototyping. - Supports heterogeneity simulation, training flow abstraction, and distributed training optimization. | - Emphasizes ease of use, deployability, and efficiency in FL experimentation and development, suitable for users with varying expertise levels. | User-friendly experimentation and prototyping in FL, suitable for research and industrial application prototypes. |
| Galaxy Federated Learning [28], [50] | - Decentralized FL framework based on blockchain technology. - Designed to train models with distributed data while maintaining data ownership and model interest. | - Aims at improving network bandwidth utilization and maintaining security under malicious node attacks, suggesting a novel approach to FL. | Addressing data privacy, high training costs, and ownership issues in AI development through a decentralized approach. |
| FLSim [30], [51] | - Domain-agnostic, scalable, and supports differential privacy, secure aggregation, and compression techniques. - Designed for simulating FL environments efficiently. | - Provides a flexible and comprehensive simulation framework for exploring various aspects of FL. | Efficiently building simulators for FL, suitable for research and development in cross-device FL. |

Based on the Table S2 in Multimedia Appendix 1, a systematic analysis reveals insightful patterns regarding the popularity, development activity, and maintenance status of the existing FL frameworks. This summary is grounded in the provided metrics: stars (indicating popularity), forks (suggesting community involvement and usage), commits (reflecting development activity), releases (showing versioning and updates), contributors (indicating collaborative effort), and maintenance status.

PySyft emerges as the most popular framework with 9.2K stars, attributed to its comprehensive approach to privacy-preserving machine learning. FATE and FedML also exhibit strong popularity, with 5.4K and 4K stars, respectively, indicating their significant impact and recognition within the community.

Regarding community engagement, PySyft leads with 2K forks, followed closely by FATE with 1.5K forks. This suggests a high level of interest and collaboration in these projects. Notably, TensorFlow Federated and FedML also show considerable engagement with 572 and 754 forks, respectively.

In terms of development activity, PySyft stands out with a remarkable 27,664 commits, indicating a highly active and evolving project. FATE follows with 13,775 commits, underscoring its robust development effort.

The contributor metric further illustrates the community and collaborative aspects of these projects. PySyft leads significantly with 421 contributors, reflecting a broad and diverse development community. Flower and TensorFlow Federated also show strong collaboration, with 120 and 106 contributors, respectively.

The Apache 2.0 license predominates among the frameworks, favored for its permissive nature, encouraging broad adoption and contribution. Exceptions include MetisFL and FLSim, which are under the BSD License, and IBM FL, which utilizes a specific license.

Maintenance status reveals a mixed landscape. While most projects are actively maintained, a few, such as FederatedScope, IBM FL, PaddleFL, and GalaxyFederatedLearning, are not recently maintained, raising considerations for potential adopters regarding long-term support and updates.

**Table S2.** Development landscape of federated learning frameworks.

| Name | License | Developed By | Stars | Forks | Commits | Releases | Contributors | Maintenance |
| --- | --- | --- | --- | --- | --- | --- | --- | --- |
| PySyft | Apache 2.0 | OpenMined | 9.2K | 2K | 27,664 | 111 | 421 | Recently Maintained |
| FATE | Apache 2.0 | Webank | 5.4K | 1.5K | 13,775 | 48 | 86 | Recently Maintained |
| FedML | Apache 2.0 | FedML | 4K | 754 | 11,726 | 6 | 66 | Recently Maintained |
| Flower | Apache 2.0 | Adap | 3.9K | 714 | 1,986 | 17 | 120 | Recently Maintained |
| TensorFlow Federated | Apache 2.0 | Google | 2.3K | 572 | 5,191 | 77 | 106 | Recently Maintained |
| FederatedScope | Apache 2.0 | Alibaba | 1.2K | 192 | 544 | 3 | 18 | Not Recently Maintained |
| OpenFL | Apache 2.0 | Intel | 644 | 172 | 606 | 9 | 77 | Recently Maintained |
| FedLab | Apache 2.0 | Smilelab | 633 | 120 | 1,422 | 9 | 13 | Recently Maintained |
| MetisFL | BSD License | BioInt | 530 | 49 | 380 | 0 | 9 | Recently Maintained |
| NVFlare | Apache 2.0 | Nvidia | 508 | 133 | 1,532 | 43 | 34 | Recently Maintained |
| IBM FL | Specific License | IBM | 473 | 135 | 178 | 10 | 11 | Not Recently Maintained |
| PaddleFL | Apache 2.0 | PaddlePaddle | 488 | 122 | 735 | 5 | 25 | Not Recently Maintained |
| FedScale | Apache 2.0 | SymbioTech | 359 | 114 | 707 | 1 | 21 | Recently Maintained |
| Plato | Apache 2.0 | Theory Lab | 304 | 71 | 1,280 | 20 | 24 | Recently Maintained |
| EasyFL | Apache 2.0 | FLGO | 409 | 78 | 1,015 | 8 | 9 | Recently Maintained |
| Galaxy Federated Learning | Apache 2.0 | GalaxyLearning | 235 | 58 | 118 | 0 | 3 | Not Recently Maintained |
| FLSim | BSD Clause License | Facebook Research | 229 | 47 | 175 | 3 | 24 | Recently Maintained |
