# Supplementary figures and images for: Accessible Ecosystem for Clinical Research (Federated Learning for Everyone): Development and Usability Study
Source: JMIR Form Res. 2024 Jul 17;8:e55496. doi: 10.2196/55496 (PMC11292148; doi:10.2196/55496)

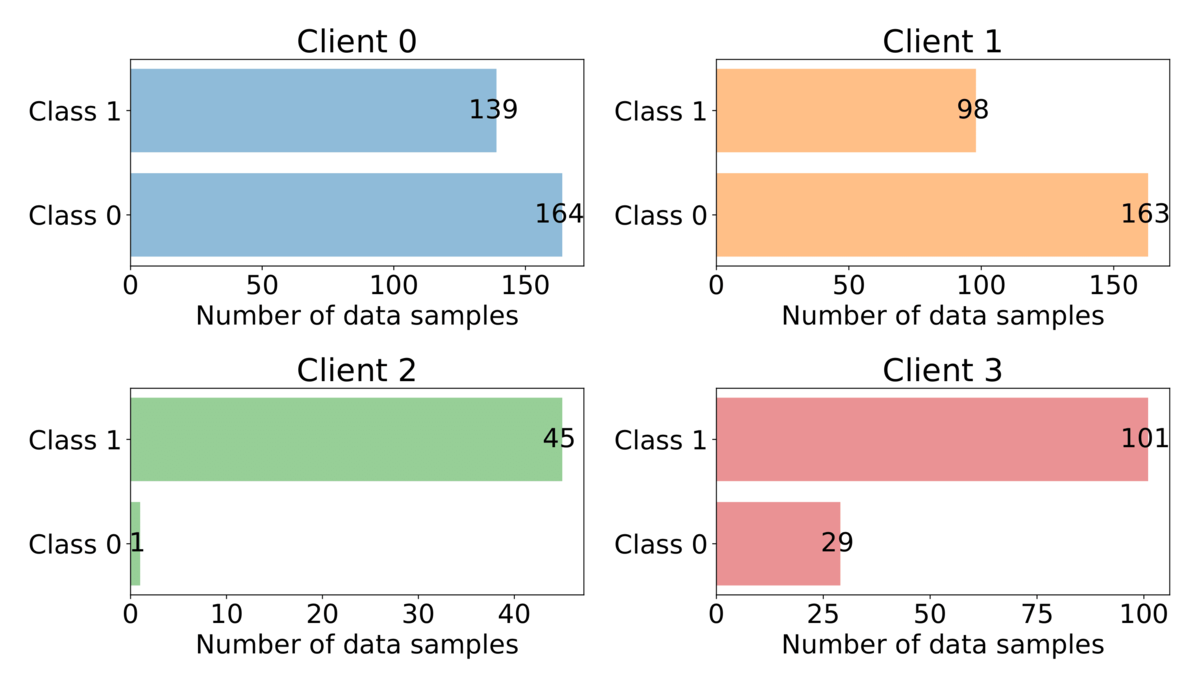

Supplement: Multimedia Appendix 2 [file formative_v8i1e55496_app2.png]
